# The structure of SeviL, a GM1b/asialo-GM1 binding R-type lectin from the mussel *Mytilisepta virgata*

**DOI:** 10.1038/s41598-020-78926-7

**Published:** 2020-12-16

**Authors:** Kenichi Kamata, Kenji Mizutani, Katsuya Takahashi, Roberta Marchetti, Alba Silipo, Christine Addy, Sam-Yong Park, Yuki Fujii, Hideaki Fujita, Tsuyoshi Konuma, Takahisa Ikegami, Yasuhiro Ozeki, Jeremy R. H. Tame

**Affiliations:** 1 Graduate School of Medical Life Science, Yokohama City University, 1-7-29 Suehiro, Yokohama, Kanagawa 230-0045 Japan; 2 Department of Chemical Sciences, Università di Napoli Federico II, Via Cintia 4, 80126 Naples, Italy; 3 Department of Pharmacy, Graduate School of Pharmaceutical Science, Nagasaki International University, 2825-7 Huis Ten Bosch, Sasebo Nagasaki, 859-3298 Japan; 4 Laboratory of Glycobiology and Marine Biochemistry, Graduate School of NanoBio Sciences, Yokohama City University, 22-2, Seto, Yokohama Kanagawa, 236-0027 Japan

**Keywords:** NMR spectroscopy, X-ray crystallography, Diagnostic markers

## Abstract

SeviL is a recently isolated lectin found to bind to the linear saccharides of the ganglioside GM1b (Neu5Ac$\alpha$(2-3)Gal$\beta$(1-3)GalNAc$\beta$(1-4)Gal$\beta$(1-4)Glc) and its precursor, asialo-GM1 (Gal$\beta$(1-3)GalNAc$\beta$(1-4)Gal$\beta$(1-4)Glc). The crystal structures of recombinant SeviL have been determined in the presence and absence of ligand. The protein belongs to the $\beta$-trefoil family, but shows only weak sequence similarity to known structures. SeviL forms a dimer in solution, with one binding site per subunit, close to the subunit interface. Molecular details of glycan recognition by SeviL in solution were analysed by ligand- and protein-based NMR techniques as well as ligand binding assays. SeviL shows no interaction with GM1 due to steric hindrance with the sialic acid branch that is absent from GM1b. This unusual specificity makes SeviL of great interest for the detection and control of certain cancer cells, and cells of the immune system, that display asialo-GM1.

## Introduction

Glycan recognition plays important roles in all forms of life, which have consequently evolved many diverse proteins (lectins) with the function of binding specifically to selected carbohydrate moieties^1^. These proteins are found to regulate such processes as development, cell growth and immune responses through their ability to detect a variety of markers at the cell surface. In mammals, for example, large families of proteins are known that bind sialic acid (Siglecs)^2^ or galactose (galectins)^3^, and these are of enormous medical interest for their roles in cell signalling and cancer. Although mammalian proteins have inevitably drawn the lion’s share of attention, marine invertebrates have also proved a rich source of lectins with novel properties that may be exploited in biotechnological or clinical applications. Many of these lectins remain uncharacterised at the protein level, but at least some of them facilitate innate immunity responses by acting as pattern recognition receptors (PRR)^4–6^. While the natural function of these proteins appears to be anti-bacterial, proliferative neoplastic disease is prevalent in marine bivalves^7^ and a number of lectins from such species have been found to induce apoptosis highly selectively in cancerous cells^8^. There is considerable interest in developing the medical potential of lectins from various sources including plants^9^. Among many others, a lectin from the edible mushroom *Boletus edulis* named BEL $\beta$-trefoil, for example, has been studied in detail^10,11^. $\beta$-trefoil lectins are found widely across species. They are generally about 150 residues in length, with internal pseudo-three-fold symmetry that is more strongly reflected at a structural level than in the sequence.

MytiLec and closely-related $\beta$-trefoil lectins from different mussels bind $\alpha$-D-galactose and N-acetylgalactosamine^12–14^. These proteins are unusual in binding a sugar ligand at each of the three sub-domains ($\alpha$, $\beta$ and $\gamma$). MytiLec is cytotoxic to certain cancer cell types, such as Burkitt’s lymphoma, that display at the cell surface the glycosphingolipid globotriose, abbreviated Gb3, which carries the trisaccharide Gal$\alpha$(1-4)Gal$\beta$(1-4)Glc^12,15^. The mytilectin subfamily shows unique sequence patterns compared to other lectins with the trefoil fold, and is of interest for its unusual specificity towards $\alpha$-linked substrates. The mytilectins were the only $\beta$-trefoil lectins known in the mytilid mussels, until the recent discovery in the purplish bifurcate mussel *Mytilisepta virgata* of a new protein called SeviL, which was purified from homogenised tissues using immobilised lactose^16^. The protein was found to cause hemagglutination of human red cells in the presence of calcium, and to bind the sugar chains of the gangliosides GM1b and asialo-GM1. Glycan array profiling of SeviL against a wide variety of oligosaccharides, 52 in all, showed no other significant interaction, apart from weak binding to SSEA-4, Neu5Ac$\alpha$(2-3)Gal$\beta$(1-3)GalNAc$\beta$(1-3)Gal$\alpha$(1-4)Gal$\beta$(1-4)Glc^16^. The first three monosaccharide residues of GM1b and SSEA-4 are identical.

Gangliosides are glycosphingolipids built from ceramide and a saccharide chain, usually carrying one or more sialic acid residues. They have numerous roles in cell signalling and signal transduction, and misregulation of their production may promote cancer^17,18^. GM1, also known as GM1a, occurs widely in mammalian tissues and performs so many essential functions that it has been called a *factotum* of signalling and regulation^19^. It is for example required for the correct functioning of tropomyosin-related kinase A (TrkA), the protein tyrosine kinase receptor for nerve growth factor (NGF) found in lipid rafts^20^. GM1 is however only one member of several series of glycolipids that differ from each other only in the addition of a single monosaccharide, and which play different biological roles. Decoding the pattern of gangliosides present at the cell surface therefore requires strict sugar binding specificity, which remains only partly explained.

Conversely, the use of saccharide biomarkers as diagnostics or for cellular control requires specific and thoroughly tested molecular tools. Asialo-GM1 is found in the membrane of mammalian natural killer (NK) cells, and in 1992 it was shown that antibodies targeting it can be used as an immunosuppressant^21^. For many years subsequently, polyclonal rabbit anti-asialo-GM1 antibodies were used to ablate NK cells in studies of innate immunity, before a lethal off-target effect was demonstrated on basophils, which also play an important role in immune responses^22^. Anti-GM1 IgG antibodies are associated with Guillain-Barré syndrome (GBS), an autoimmune-mediated neuropathy, but these human proteins also show a broad range of reactivity with other gangliosides^23^. Although commercial antibodies have played an important role in molecular biology, recent tests of such preparations have found some alarming results^24^. Even widely-used products have been found to give false positive or false negative results, leading to a drive towards validation, using gene knock-outs where possible. Lectins provide an obvious alternative solution to the problem of detecting target saccharides, if a protein of suitable specificity can be found. Such proteins offer tremendous cost savings if they can be produced in bacteria, and as pure proteins they allow much more precise characterisation than a polyclonal antibody.

The natural function of SeviL remains unknown, but if SeviL is added to mammalian carcinoma cells expressing asialo-GM1 then it activates apoptotic pathways, causing cell death^16^. The 129 residue sequence of SeviL is unrelated to the 149 residue MytiLec, but shows three weakly-conserved tandem repeats of about 40 residues, each with a tryptophan residue at an equivalent position. This is highly suggestive that SeviL resembles the classical R-type lectin structure, with a QxW motif in each of the three subdomains^25^. The lack of overall sequence similarity, especially around the possible ligand binding sites, and the small size of the protein relative to other $\beta$-trefoils, make it difficult to produce a reliable homology model. In order to understand the ligand binding specificity of SeviL, we have solved the crystal structure of the protein in both apo and substrate-bound forms, and confirmed the binding site by solution state NMR. The binding of different ligands was also measured by calorimetry, allowing us to correlate the structural model with the ligand specificity of the protein.

## Results

### Overall structure

**Figure 1:**
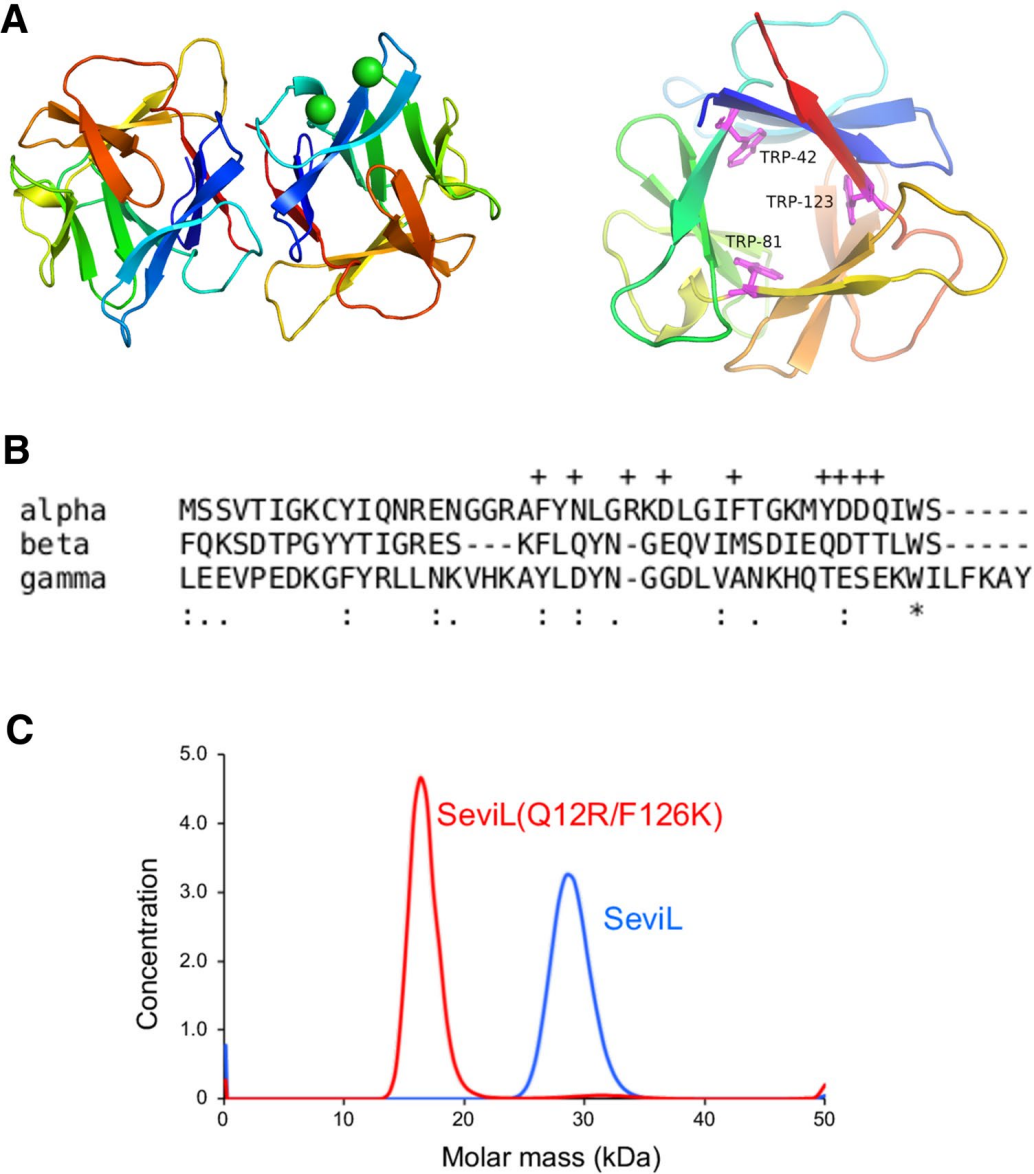
(**A**) The C$\alpha$ trace of the apo-SeviL dimer, looking along the dimer symmetry axis. Each subunit is coloured from blue (N-terminus) to red (C-terminus), with $\beta$-strands shown as arrows. There is no helical secondary structure in the model. Chloride ions (associated with the B subunit) are shown as green spheres. A separate view of a single subunit is shown on the right, looking along the pseudo-symmetry of the $\beta$-trefoil fold. The three tryptophan residues are shown as pink sticks, and reflect the internal symmetry of the protein chain. Structural figures were drawn using PYMOL^26^, which was used to detect secondary structure automatically. (**B**) A sequence alignment of the three subdomains of SeviL. Crosses over the sequence indicate the residues in the $\alpha$ subdomain that contact the sugar ligand. Colons indicate conservation of residue type, and the asterisk shows the only residue conserved in each subdomain, the tryptophan. (**C**) Molecular weight estimation by sedimentation velocity experiment as described in Methods/Analytical Ultracentrifugation. Wild-type SeviL shows a mass corresponding to the dimer, but the double mutant SeviL(Q12R/F126K) is almost entirely monomeric. A high rotation speed (50,000 rpm) was used to give better separation of species by mass, so the arbitrary concentration units reflect the presence of monomer and dimer.

Gene cloning, protein expression and crystallisation were carried out as described in Supplementary Information. Apo-SeviL crystallised in space-group $P32_1$, and diffracted to 1.7 Å resolution. Searching the PDB for known models with significant sequence similarity to SeviL yielded only one protein, the BEL $\beta$-trefoil (PDB 4I40)^10^. The structure was solved by molecular replacement using the program BALBES^27^. Crystallographic data and refinement statistics are given in Table 1. Two monomers (A and B) of SeviL are found in the asymmetric unit, revealing a $\beta$-trefoil fold consisting of three sub-domains (Fig. 1A). The structure is well ordered, but several N-terminal residues (up to Val 4) are not visible in the 2mFo-DFc electron density map of one subunit (A). The entire polypeptide chain of the B subunit is ordered, including N-terminal residues that remain after removal of the histidine tag by thrombin. These residues lie close to a crystal contact however, so it is likely that they are flexible in solution. The model shows expected geometrical features and has no Ramachandran outliers. No non-crystallographic symmetry restraints were applied, but least-squares fitting the two protein chains by matching the 121 C$\alpha$ atoms of residues 9–129 (the C-terminus) gave an rmsd of 0.36 Å. The two copies of Thr 33 show different positions in the Ramachandran plot, one having an $\alpha$-like configuration and the other $\beta$-like. This local asymmetry seems to be due to the fact the carbonyl oxygen of Thr 33 in one subunit hydrogen bonds with water molecules, deep within the protein dimer interface, that are close to the carbonyl atoms of Asn 16 in both subunits. These water molecules are unable to accept additional hydrogen bonds, forcing the other Thr 33 residue to take up a different conformation, allowing its carbonyl to interact with a surface solvent molecule. The same feature is found in both models of the protein, with and without ligand (SFigure 1).

**Table 1:** ^a^Rmerge = $\Sigma I_i - \langle I \rangle$ / $\Sigma I_i$, where $I_i$ is the intensity of an observation and $\langle I \rangle$ is the mean value for this reflection, and the summations are over all reflections. Values in parentheses are for the highest resolution shell. ^b^R factor is $\Sigma _h ||Fo(h)| - |Fc(h)|| / \Sigma _h Fo(h)$, where *Fo* and *Fc* are the observed and calculated structure factor amplitudes, respectively. The free R factor was calculated with 5% of reflections omitted from the refinement.

**Data collection statistics**		
Data-set	Apo	asialo-GM1 complex
Space group	*P*32_1_	*P*4_1_2_1_2
Wavelength (Å)	0.98	0.98
Unit cell (Å)	a = 86.2, b = 86.2	a = 61.6, b = 61.6
	c = 67.6	c = 136.8
Resolution range (overall/outer shell)	43.1–1.70/1.73–1.70	43.6–1.6/1.63–1.60
Reflections (measured/unique)	371,509/31,674	725,030/35,352
Completeness (overall/outer shell, %)	98.3/97.1	99.7/98.9
^a^R$_{merge}$ (overall/outer shell, %), Rpim	8.7/127.1/3.8	19.8/320.5/5.0
Multiplicity (overall)	11.7	20.4
Average $I/\sigma (I)$ (overall/outer shell)	5.1/0.5	25.1/2.3
**Refinement statistics**		
	Apo (PDB 6LF1)	aGM1-bound (PDB 6LF2)
Resolution range (Å)	43.1–1.70	34.2–1.6
^b^R-factor/free R-factor (%)	19.3/22.8	19.5/22.8
Rmsd bond lengths (Å)/angles (°)	0.005/0.74	0.006/0.80
No. of water molecules	113	138
Average B factors (Å^2^) (protein/water/ligand)	30.2/31.1/–	14.8/19.3/19.0
% residues with favoured Ramachandran angles	100.0	100.0
% residues with outlier Ramachandran angles	0	0

The $\beta$-trefoil fold shows pseudo-three-fold rotational symmetry, with three subdomains of the protein (called $\alpha$, $\beta$ and $\gamma$) that each contain four $\beta$-strands. In the case of SeviL the internal symmetry is poorly reflected in the sequence (Fig. 1B), although each subdomain contains an equivalent tryptophan residue, as found in other members of the family.

**Figure 2:**
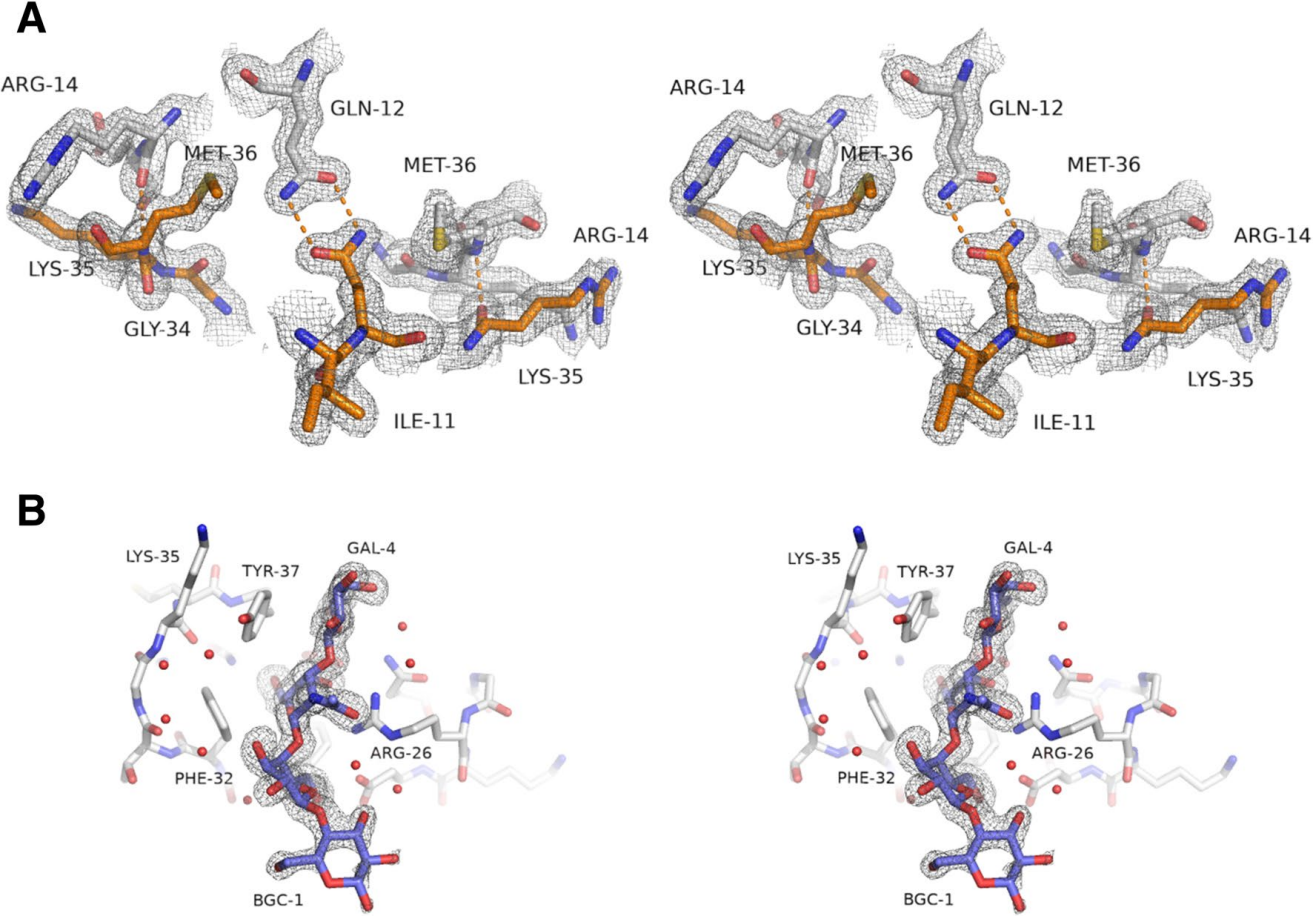
(**A**) Stereo view of the 2mFo-DFc electron density map of apo-SeviL, showing the dimer interface created by the two-fold symmetry axis. Carbon atoms are shown in brown for one subunit and white for the other. Oxygen atoms are coloured red and nitrogen blue. Hydrogen bonds are shown as orange dotted lines. The Gln 12 side-chain forms a double hydrogen bond with its symmetry mate. The nitrogen atom of Met 36 donates a hydrogen bond to the oxygen atom of Arg 14 in the partner subunit. The map is contoured at 1 $\sigma$. (**B**) The 2mFo-DFc electron density map covering the tetrasaccharide bound by SeviL. The glucose residue is more mobile, but the temperature factors and density for the other residues indicate high occupancy in a single conformation.

The two monomers in the dimer are closely associated by hydrophobic interactions and hydrogen bonds involving 11 residues on each subunit. Gln 12 and Phe 126 lie close together, and their side-chains lie opposite their symmetry mates at the dimer interface. Based on the crystal structure, a double mutant was designed (Q12R/F126K) that was expected to fold in a monomeric form, missing important contacts at the dimer interface. The mutant protein proved stable enough to be expressed at a high level, and purified in the same way as the native protein. Analytical ultracentrifugation confirms the native protein is a tightly-bound dimer in solution, but the dimer is almost undetectable for the Q12R/F126K mutant (Fig. 1C). Met 36 (and its symmetry mate) contributes the largest contribution of buried surface area of any residue at the interface, becoming entirely sequestered from the bulk solvent (Fig. 2A). The buried interface area between SeviL monomers is 780 Å^2^, but too small for PISA^28^ to predict a stable dimer with confidence. Contacts between the two subunits are listed in STable 1. Compared to MytiLec, SeviL is truncated at the C-terminus by 13 residues. These 13 residues form part of the dimer interface in MytiLec, so the two subunits of SeviL are held together by a quite different set of interactions. $\beta$-trefoil lectins are often found to be dimeric, but the dimer interactions differ widely in buried surface area and orientation^29^.

Within the apo crystal structure, a crystal contact is formed with Arg 26 of the B chain approaching within 3.6 Å of its symmetry mate. Two nearby balls of electron density were modelled as chloride ions, which would counter the repulsive charge on the two residues (SFigure 2). One chloride ion sits in a hydrophobic pocket formed by Phe 21, Phe 32 and Tyr 37; it comes within 3 Å of the side-chain of Asp 39, which also lies close to Arg 26 from the neighbouring molecule. No chloride ions were modelled near the A subunit, and it appears this interaction is unique to the crystal form, which was crystallised at pH 3.5.

### Comparison with other trefoil lectins

Although SeviL shows only weak sequence similarity to known structural models, using DALI^30^ to find structural homologues in PDB with the model of the Sevil subunit yielded a number of significant matches. The closest (with a Z-score of 18.2) is lectin LSL from the mushroom *Laetiporus sulphureus* (PDB 1W3A)^31^. This is a hexameric pore-forming lectin with an N-terminal $\beta$-trefoil domain. The $\gamma$ subdomain binds the non-reducing galactose (Gal) moiety of lactose. A sequence alignment of SeviL and its closest structural homologues is given in SFigure 3. DALI identified 24 structures with a Z-score of 15 or higher, which are mainly listed as lectins or proteins of unknown function. Several are reported to be insecticidal and one is actinohivin, a gp120-binding lectin that is reported to block HIV infection^32^. Strong structural similarity is also seen to the lectin domain of interleukin-33 (PDB 5VI4)^33^, an agonist cytokine that is mainly expressed by epithelial, endothelial and fibroblast cells, and an attractive therapeutic target^34^. Overall however, the sequence and structure of SeviL provide few clues to its biological function.

**Figure 3:**
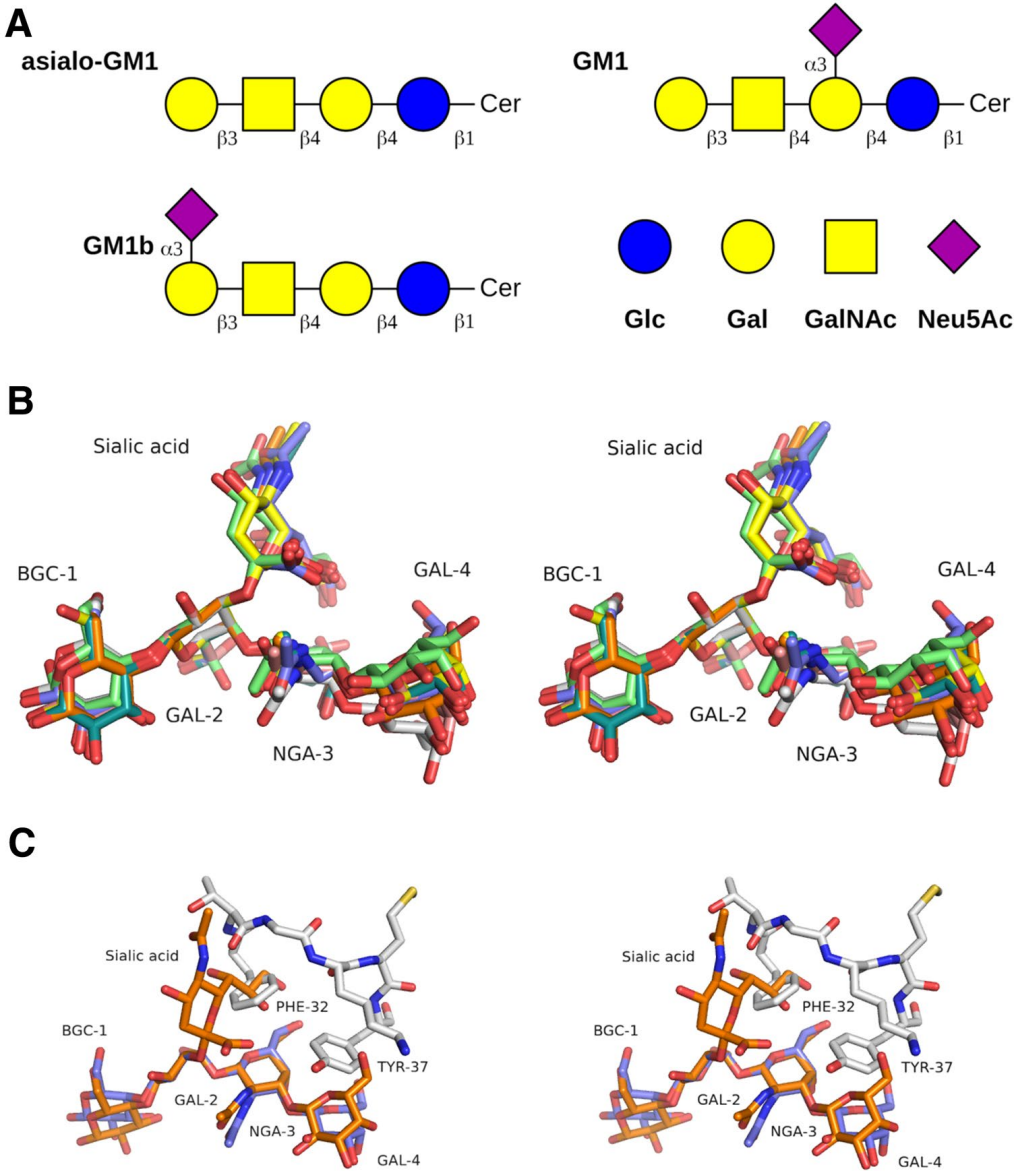
(**A**) The structure of the related glycosphingolipids GM1, GM1b and asialo-GM1. The glucose is linked in each case to ceramide. (**B**) A stereo view of the least-squares overlay of the tetrasaccharide bound to SeviL and the GM1 saccharide found in models from PDB of different lectin complexes. Each saccharide was overlaid by least-squares fit of ten atoms of the Gal-2 residue (omitting O1 and O6). Carbon atoms of asialo-GM1 bound to SeviL are shown in white. Carbon atoms of other complexes are shown with the colours green (endoglycoceramidase I, PDB 5j7z)^35^, orange (polyomavirus genotype 3 VP1, PDB 4x0z)^36^, purple (polyomavirus VP1, PDB 4u60)^37^, dark green (ECX AB5 holotoxin, PDB 4l6t)^38^, pink (*Agrocybe aegerita* lectin AAL, PDB 3m3q)^39^, yellow (cholera toxin B-pentamer, PDB 3chb)^40^. (**C**) A stereo view showing the saccharide of GM1 modelled into the binding site of SeviL by overlay of the Gal-2 residue. The sialic acid residue of GM1 clashes strongly with Phe 32 of the protein.

### Sugar binding sites

The saccharides of asialo-GM1 (Gal$\beta$(1-3)GalNAc$\beta$(1-4)Gal$\beta$(1-4)Glc) and GM1b (Neu5Ac$\alpha$(2-3)Gal$\beta$(1-3)GalNAc$\beta$(1-4)Gal$\beta$(1-4)Glc) are commercially available, and shown schematically in Fig. 3A. It was attempted to crystallise SeviL with each ligand, but crystals suitable for diffraction data collection were only produced with asialo-GM1. These crystals, in space-group $P4_12_12$, allowed data collection to 1.6 Å resolution (Table 1). An initial model was built using molecular replacement with the apo structure, revealing a dimer in the asymmetric unit, with identical subunit interface. The data allowed the sugar ligands to be placed unambiguously at one site per polypeptide, close to this interface but with each saccharide bonded exclusively by one protein subunit. Like the apo model, the final complex structure has no Ramachandran outliers. The electron density map covering the ligand is shown in Fig. 2B. Comparing the main chain atoms of a single subunit, the apo and liganded forms show a rmsd of up to 0.36 Å, depending on which subunits are compared, indicating that the protein shows no movement on ligand binding. Crystal contacts in the different space-groups also do not apparently distort the protein significantly. Least-squares comparison of the dimer in the apo and liganded models gives a rmsd of 121 C$\alpha$ atoms (residues 9–129) of 0.53 Å. The largest structural change between the two models is found at the side-chain of Arg 26, which moves to engage the saccharide. There is no suggestion of domain shifts indicative of allostery. Comparing the two subunits of the liganded model gives an rmsd of 0.36 Å. The sugar shows a significant shift in the position of the glucose residue between the two copies in the asymmetric unit due to crystal contacts. Smaller shifts in other residues of the sugar are related to the asymmetry of Thr 33, which touches the side-chain of Phe 32, but the contacts between the protein and ligand are essentially the same in both subunits.

Two copies of the tetrasaccharide ligand are observed in the asymmetric unit, and each hexose unit is clearly visible in the electron density map. The glucose (BGC-1) appears to be more flexible, making no direct contact with the protein and having weaker electron density than the remainder of the ligand. For one copy of the saccharide in the asymmetric unit, the glucose residue makes slight crystal contacts which possibly reduce its movement within the crystal lattice, but apparently have little effect on the overall ligand conformation (see below). The sugar binding site is centred around the position of one of the chloride ions identified in the apo-model, with the N-acetylgalactose residue (NGA-3) displacing the halide (and the Arg 26 side-chain of a neighboring protein molecule) from the pocket formed by Phe 21, Phe 32 and Tyr 37. Contacts between asialo-GM1 and SeviL are listed in STable 2. Binding of asialo-GM1 to a subunit of SeviL buries 250 Å^2^ of the protein surface area, and forms eight hydrogen bonds between the protein and the saccharide. The carboxyl side-chains of Asp 28 and Asp 38 form hydrogen bonds with the O6 atoms of Gal 2 and Gal 4 respectively. Asp 39 makes much more extensive ligand interactions, including hydrogen bonds with O4 and O6 of the N-acetylgalactose moeity (NGA-3), as well as lying close (3.0 Å) to O6 of Gal 4. The side-chain of Arg 26 approaches within 3.6 Å the O3, O4 and O5 atoms of NGA-3, forming two hydrogen bonds with O4. A single hydrogen bond to the ligand involves the main-chain of SeviL, with Asp39 donating a bond to O6 of Gal4 through its nitrogen atom. On the opposite side of the ligand from Arg 26 and Asp 28, the aromatic side-chains of Phe 32 and Tyr 37 lie close together, providing a hydrophobic surface that touches each hexose except the glucose residue. The N-acetyl group makes no strong interaction with the protein, pointing into the bulk solvent, suggesting it plays no part in ligand specificity.

The structures of a number of complexes of lectins and GM1 have been determined crystallographically, allowing us to compare the different protein-sugar interactions. An in-house program called CFIT (JRHT, unpublished) was used to fit by least-squares the GAL-2 residue of each complex with that of asialo-GM1 bound to SeviL. Overlap of the different ligands shows that, despite the very different binding modes, the saccharides of each complex show remarkably similar conformations (Fig. 3B). Comparison by NMR of GM1 and asialo-GM1 at membrane surfaces has previously shown that the additional sialic acid of GM1 group has little effect on the order or conformation of the surprisingly stiff saccharide backbone, with torsion angles no more than 10° different between the two gangliosides^41^. Even though the glucose residue forms fewer contacts with the proteins, and has weaker electron density in the final 2mFo-DFc map, it appears to be little more variable in conformation than the other residues of the tetrasaccharide. No torsional restraints were applied on the bonds between saccharide residues in the refinement. Modelling the GM1 saccharide into the binding site of SeviL (based on the known complex structures) shows that the sialic acid group connected to GAL-2 would cause a severe steric clash with Phe 32 (Fig. 3C).

Lectins are often found to bind their substrate sugars specifically by recognizing the water structure around the ligand, so that, for example, the recognition of the Thomsen-Friedenreich (TF) antigen (Gal$\beta$(1-3)GalNAc$\alpha$1-Ser/Thr) by the lectin AAL from the mushroom *Agrocybe aegerita* is found to involve a hydrogen bond network formed by two water molecules held between the saccharide and arginine and glutamate side-chains^39^. The human protein galectin-3 also binds TF antigen and related sugars including the GM1 saccharide, with arginine and glutamate residues contacting the ligands indirectly through water molecules in a similar fashion to AAL^42^. SeviL is notably different, despite binding very similar ligands, in that no water molecules are found between the protein and bound asialo-GM1 saccharide. This close association gives SeviL a narrower range of binding partners than galectin-3, without apparently giving a significantly greater affinity of binding^42^.

### NMR

**Figure 4:**
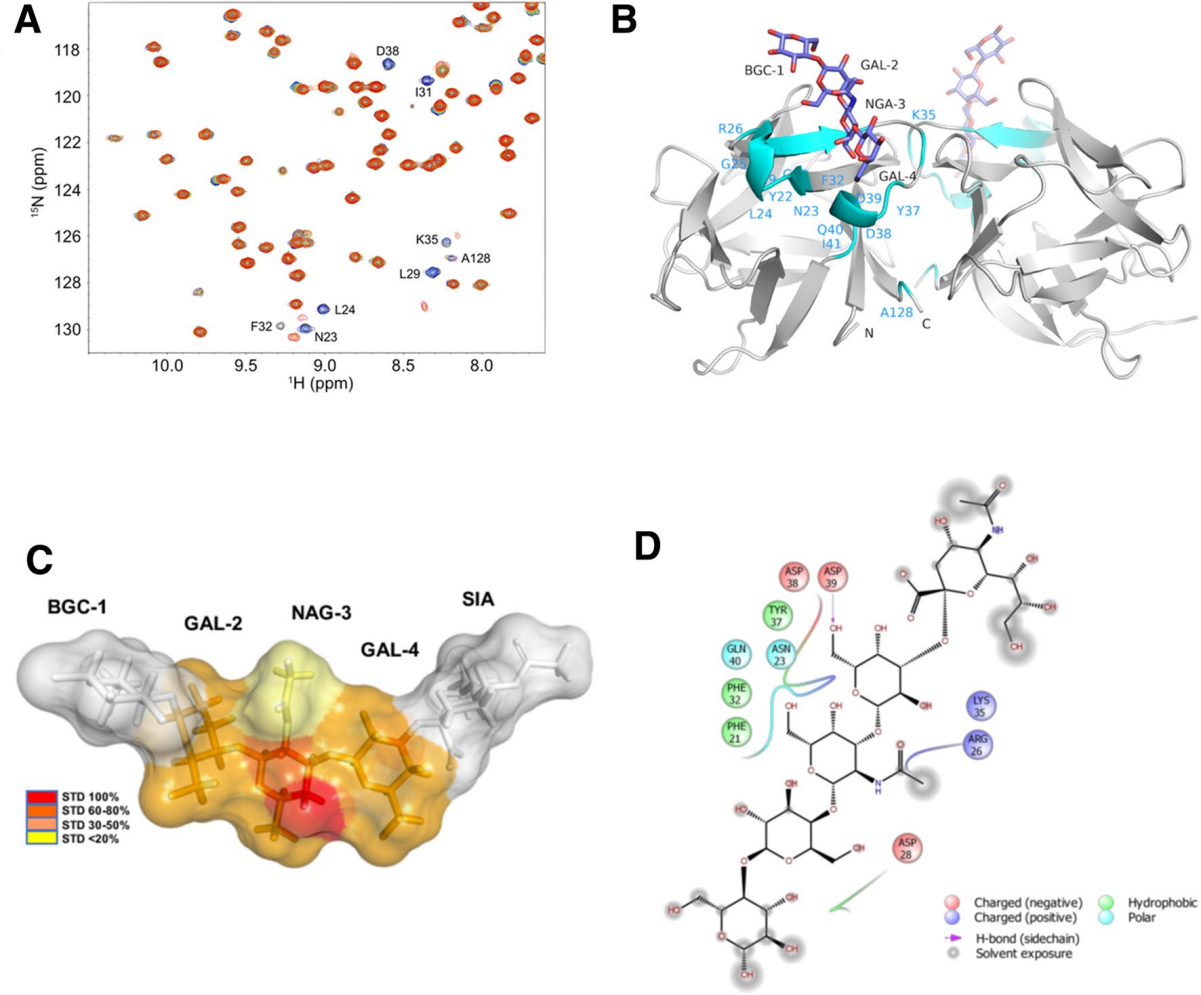
(**A**) Central region of the 2D ^1^H-^15^N HSQC spectra of SeviL, showing residues affected by the presence of asialo-GM1. Spectra are coloured by concentration of asialo-GM1 saccharide: 0 mM (black), 0.05 mM (blue), 0.1 mM (cyan), 0.2 mM (green), 0.3 mM (yellow), 0.5 mM (orange) and 1 mM (red). (**B**) The ribbon diagram of the SeviL dimer, showing the bound saccharide. Residues showing altered HSQC signals in the presence of asialo-GM1 saccharide are coloured blue. (**C**) Epitope mapping of interactions with GM1b saccharide. The molecular model of the sugar is shown, with the van der Waals surface coloured by STD signal. The residues at each end of the saccharide showed no significant bonding with SeviL. (**D**) Diagram showing the residues surrounding the sugar ligand. The glucose (bottom left) and sialic acid (top right) residues are highly solvated and make no strong bonds with the protein.

In order to observe the effects of saccharide binding to SeviL in solution, the HSQC spectrum of ^15^N-labelled protein was fully assigned, and measured in the absence and presence of the asialo-GM1 saccharide (SFigures 4 and 5). The ^1^H, ^15^N and ^13^C chemical shift values of the SeviL backbone have been registered in BMRB (http://www.bmrb.wisc.edu/) with access code 26300. Peaks showing notable shifts with added ligand are shown in Fig. 4A. While the majority of residues show no influence of ligand on their environment, a small selection show small but distinct changes. Mapping these residues on the crystallographic model shows a perfect correlation with the binding site, although several residues are distant from the ligand and influenced indirectly (Fig. 4B). Ala 128 for example lies almost 13 Å from the ligand, but the C-terminal tail of the protein is flexible, and Ala 128 touches Ile 41, adjacent to Asp 39 and Gln 40, which both bind the saccharide directly. Overall the HSQC spectra indicate a slight tightening of the residues at the binding pocket, but show no changes across the dimer interface that suggest significant allosteric interaction.

Saturation transfer difference (STD) NMR experiments^43^ were performed on the protein-ligand mixture to gain further molecular insights into the recognition of GM1b saccharide by Sevil (see Methods/NMR Analysis). As described in the supporting information (see SFigures 6 and 7), dramatic changes were observed in the relative intensity of STD signals with respect to the reference spectrum, indicating specific binding (SFigure 8). Significant STD effects were detected for the GalNAc and Gal residues of GM1b, although weaker for GAL-2 than NGA-3 or GAL-4. No evidence was found of any interaction of the protein with the glucose or sialic acid residues, which remained solvent exposed in the complex (Fig. 4C,D). The X-ray structure was used to model GM1b binding to SeviL, and theoretical STD values were determined using CORCEMA-ST (complete relaxation and conformational exchange matrix calculations), showing an excellent fit (NOE R-factor 0.3) to the experimental values (SFigure 9), further supporting the key role of the central NGA-3 and GAL-4 residues in binding.

### Ligand binding assays

**Figure 5:**
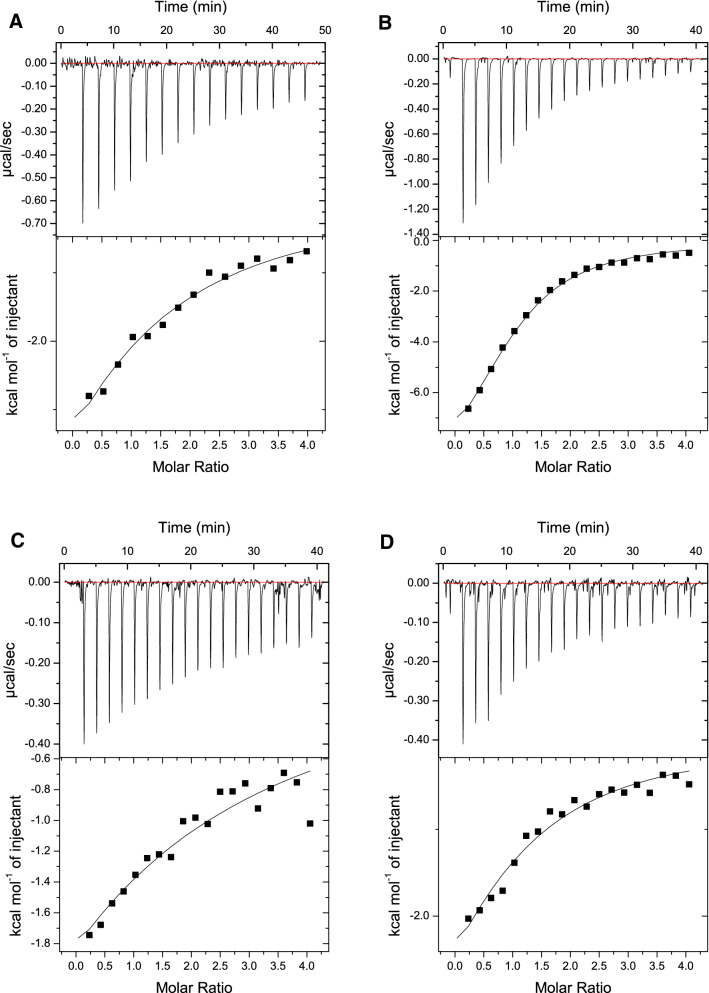
(**A**) A thermogram produced by injecting 1 mM asialo-GM1 saccharide into 50 $\upmu$M SeviL. The experiment was repeated three times, each thermogram being fitted to a one-site model (shown as a black line). Dissociation constants were calculated as weighted averages, and errors were calculated as weighted standard deviations, using the reciprocal chi-square values of each individual fit as weights. The $K_d$ was determined to be 0.1 mM ± 32 $\upmu$M. (**B**) A similar result to that shown in (A), but produced using 1 mM GM1b as ligand. $K_d$ = 0.1 mM ± 92 $\upmu$M. (**C**) 1 mM asialo-GM1 titrated into 50 $\upmu$M SeviL (Q12R/F126K). $K_d$ = 0.1 mM ± 36 $\upmu$M. (**D**) 1 mM GM1b titrated into 50 $\upmu$M SeviL (Q12R/F126K). $K_d$ = 0.1 mM ± 29 $\upmu$M.

To confirm the binding specificities implied by the structural model, ITC was used to measure the affinity of SeviL for different small sugar ligands, fitting the binding curve to a simple model with one type of independent site. Experimental details are given in Methods/Isothermal Titration Calorimetry. Glucose was not seen to interact with the protein at all, but galactose, N-acetylgalactose and lactose were found to bind with $K_d$ values between of 8 mM and 12 mM (data not shown). Asialo-GM1 and GM1b were found to bind SeviL more tightly, but binding was not highly exothermic (Fig. 5A,B). ITC experiments were repeated with the monomer mutant SeviL (Q12R/F126K). Again the affinity and heat of binding were modest, making accurate parameter determination difficult, especially for the heat of binding (Fig. 5C,D). Errors in $K_d$ for the native protein and monomer were determined from triplicate experiments. To produce a stronger signal and lower noise, further experiments were attempted with the concentrations of the native protein and ligand increased ten-fold. At high concentrations the saccharide moieties of asialo-GM1 and GM1b were found to produce thermograms with a very different shape, with an initial marked increase in the heat release per mole of injectant as the binding reaction proceeded (SFigure 10). Such thermograms are not unknown among protein homo-oligomers with one binding site per subunit^44^. By assuming a model of non-interacting sites the data for both ligands can be interpreted in terms of an exothermic and an endothermic binding site, with $K_d$s of roughly 0.1 mM each, but it seems more likely that self-interaction of the saccharides is responsible for this effect. The monomer protein could not be concentrated without indications of aggregation, which prevented ITC experiments under such conditions. Overall the calorimetric data provided no evidence for a significant degree of cooperativity between the binding sites, which retain their affinity and specificity in the monomer protein. The thermograms for asialo-GM1 and GM1b suggest the sialic acid residue of the latter ligand makes little contribution to the affinity of SeviL binding. These data confirm the NMR results obtained with the complex formed by GM1b and Sevil, which clearly indicated that the terminal Neu5Ac residue did not significantly contribute to the interaction with the protein. This is in agreement with the crystallographic model, which shows the O3 atom of the terminal galactose residue of asialo-GM1 points directly away from the protein, so that a sugar residue attached at this point would make no direct interaction with SeviL, leaving the affinity little changed. Different stereo views of the interaction between protein and ligand are shown in SFigure 11.

### Hemagglutination activity

**Figure 6:**
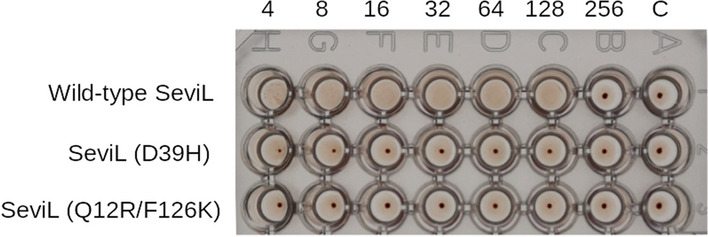
Hemagglutination of red blood cells by SeviL. 20 $\upmu$L of a two-fold dilution of SeviL (initially 10 mg/mL) were serially diluted and added to the same volume of a red cell suspension containing 0.25% Triton-X100 and 10 mM calcium chloride, and incubated for 1 h at room temperature. Appearance of a dot indicates no agglutination. The number over the wells indicates the protein dilution factor. Top row: dimer SeviL, middle row: dimer SeviL (D39H), and bottom row: monomer SeviL (Q12R/F126K). Column C is a negative control with no added lectin.

SeviL is able to hemagglutinate red cells in the presence of calcium^16^. Mutants of SeviL, designed on the basis of the model described here, that are unable to bind the saccharide or form the dimer do not hemagglutinate (Fig. 6). The band 3 protein of red blood cells exposes many N-acetyllactosamine units (Gal$\beta$(1-4)GlcNAc) at the cell surface^45^, which would provide a means of binding SeviL, but the requirement for calcium remains unexplained. The crystal structure of the protein offers no potential calcium binding site on SeviL itself, and both ITC and NMR analyses showed that calcium is not required for saccharide binding.

## Discussion

Gangliosides, a sub-group of glycosphingolipids, are known to be involved in a wide variety of physiological processes, including cell growth and migration, as well as apoptosis^46,47^. The use of genetically-engineered mice has highlighted their importance to proper development of the nervous system^48,49^, where they also play essential roles in maintenance and repair^50^. Although many different gangliosides are found throughout the body, four closely-related types (GM1, GD1a, GD1b, and GT1b) are found to be roughly equally present in the adult mammalian brain, where between them they account for all but a few percent of the total gangliosides present^51^. Gangliosides are the receptors for viruses (such as SV40) and toxins (such as cholera toxin)^52,53^. The majority of cancer cells show some form of aberrent glycosylation of glycolipids at the cell surface, and this has led to considerable interest in the development of immunotherapies^54,55^. Asialo-GM1 for example is expressed on bone metastatic C4-2B prostate cancer cells, and appears to play a significant role in the progression of the disease through its interaction with the integrin $\alpha 2\beta 1$ receptor, which maintains the invasive phenotype^56^.

SeviL is a recently described lectin, of uncertain biological function, isolated from mussels. Here we have shown the protein is a dimeric member of the well-known $\beta$-trefoil superfamily. The fold is defined by the pseudo-three-fold symmetry axis that relates six beta-hairpins, three of which form a truncated barrel-like structure, and three of which form a cap. PFAM (https://pfam.xfam.org) defines 23 different subfamilies with this fold, including ricin-type lectin and fibroblast growth factor. $\beta$-trefoil lectins are also known as R-type carbohydrate recognition domains (CRDs) after ricin, the toxin from castor beans, but surprisingly PFAM fails to recognise proteins such as MytiLec as members of the family, apparently because of the highly unusual sequence, which includes no tryptophan residues^29^. The CAZY database of carbohydrate-binding modules (www.cazy.org) also fails to include MytiLec in the family CBM13, which is defined as $\beta$-trefoil lectins. Several $\beta$-trefoil lectins are known that have growth suppression activity against microorganisms or cancerous cells^10,57^. Although the fold is common, lectins in this family are generally found to accommodate galactose or simple derivatives of it in the binding site. SeviL clearly forms a $\beta$-trefoil structure that can be recognised as such by DALI, but it is unusual in having a larger binding site that forms hydrogen bonds with three hexose residues in its ligand. Through a combination of structural analysis, sequence comparison and manual search we found that SeviL shows significant sequence similarity to actinohivin, a lectin of interest as a possible means of controlling HIV infection, and a substrate-capture domain within a xylanase from *Streptomyces olivaceoviridis*^58^; sequence and structural comparisons produced with Espript (http://espript.ibcp.fr)^59^ are shown in SFigure 3. Both of these proteins show no similarity to SeviL at the asialo-GM1 binding site, and their substrate preferences are completely different. Notably both proteins preserve the classic QxW motif in each subdomain, whereas SeviL only has this motif in the $\alpha$ subdomain.

SeviL is the first lectin to be described that shows an exquisite selectivity for asialo-GM1 over the closely related GM1, suggesting it may well be a useful tool in ganglioside research, and especially in studies of NK cells, which are currently of enormous interest due to their central role in the control of cancer by the immune system. Further work is needed to investigate the biological function of SeviL, and whether it may be used to control the activity of cancer cells, or the immune cells that attack them. We have shown (using the assay described in Methods) that SeviL can trigger hemagglutination at low concentration, but only in the presence of calcium. The structure shows no evidence of any calcium binding site, such as a cluster of carboxylate bearing side-chains, indicating that the requirement for calcium is due to a secondary effect on the target cell. Eliminating saccharide binding with a single site mutation, changing Asp 39 to histidine, abolishes hemagglutination. Hemagglutination is also lost in a SeviL mutant that retains sugar binding, but forms only monomers. The dimeric form therefore appears to be required for at least some of the biological effects of the native protein, which include stimulation of apoptotic pathways in cells that SeviL can bind^16^. The models described in this paper form the basis of further work to develop a new biosensor for asialo-GM1, a useful addition to present lectin arrays, as well as understand the biological effects of SeviL on cells that display its target saccharide.

## Methods

### Analytical ultracentrifugation

The sample concentration was estimated as 1.0 $\upmu$g ml$^{-1}$ from absorbance at 280 nm. Sedimentation velocity experiments were carried out using an Optima XL-I analytical ultracentrifuge (Beckman-Coulter) using an An-50 Ti rotor. Cells with a standard Epon two-channel centre-piece and sapphire windows were used. 400 $\upmu$L of the sample and 420 $\upmu$L of the reference solution (50 mM potassium phosphate pH 6.8 and 0.1 M NaCl) were loaded into the cell. The rotor was kept stationary at 293 K in the vacuum chamber for 1 h prior to each run fror temperature equilibration. Absorbance at 280 nm scans were collected at 10 min. intervals during sedimentation at 50,000 rpm. The resulting scans were analysed using the continuous distribution *c*(*s*) analysis module in the program SEDFIT^60^. Frictional ratio (f/fo) was allowed to float during fitting. The *c*(*s*) distribution was converted into a molar mass distribution *c*(*M*). Partial specific volume of the protein, solvent density, and solvent viscosity were calculated from standard tables using the program SEDNTERP^61^.

### Isothermal titration calorimetry

Experiments were performed using a MicroCal iTC200 (GE) instrument. The sample of SeviL (in 50 mM phosphate buffer, pH 6.8, 0.1 M NaCl) was placed in the cell, and equilibrated at 298 K. The selected ligand was dissolved in the same buffer, and injected into the protein sample (with 20 injections of 10 $\upmu$L each), allowing the baseline to stabilise between injections. The data were analysed using the manufacturer’s software, assuming a simple single site model, to yield a $K_d$ value and chi-square value from each experiment. 50 $\upmu$M SeviL was mixed with galactose, N-acetylgalactosamine and lactose at 50 mM concentration, whereas T-antigen concentration was 15 mM. Asialo-GM1 and GM1b were used at 1 mM concentration, each experiment being repeated three times, and errors were determined as the weighted standard deviation of these three values. Higher concentrations of protein and ligand were used in the case of these longer ligands. 10 mM asialo-GM1 was injected into 1 mM SeviL, and 12 mM GM1b was injected into 0.8 mM SeviL.

### Crystallisation and structure determination

Crystallisation of apo-SeviL was performed at 293 K using the hanging-drop vapor diffusion method. Crystals grew in 0.1 M citric acid, pH 3.5, 3 M NaCl. Co-crystals of SeviL and asialo-GM1 were grown using 0.1 M MIB, pH 5.0, 25% PEG 1500. Crystals of both types were washed briefly in mother liquor containing 15% glycerol as cryo-protectant before being stored in liquid nitrogen.

Data were collected at beam-line 17A of the Photon Factory, Tsukuba, using incident radiation of 0.98 Å wavelength. A total of 420 images of 0.5$^\circ$ oscillation were collected for the apo dataset, and 720 images for the sugar complex. Data processing and scaling were carried out with HKL2000 and SCALEPACK^62^. The space-group of the apo form was found to be *P*32_1_, and that of the complex $P4_12_12$. Both crystal forms have two molecules in the asymmetric unit. Data manipulation was performed throughout using the CCP4 package^63^. The complex structure was determined by molecular replacement from the apo model, and refined in a similar manner. Two molecules of asialo-GM1 were found at pseudo-equivalent positions in the asymmetric unit of the complex structure. Model manipulation was carried out with COOT^64^. Default restraints were applied for the protein and saccharide during model refinement with PHENIX^65^. Data collection and refinement statistics for both models are shown in Table 1.

### NMR analysis

SeviL labelled uniformly with $^{15}$N was expressed by culturing the bacteria in M9 minimal medium containing 4.0 g/L D-glucose with 1.0 g/L $^{15}$NH_4_Cl as sole nitrogen source. For SeviL labelled with $^2$H, $^{13}$C, and $^{15}$N, the bacteria were cultured in a D_2_O M9 minimal medium containing 2.0 g/L [$^2$H, $^{13}$C]-D-glucose and 1.0 g/L $^{15}$NH_4_Cl. The proteins were purified as described above. All protein-based NMR experiments were performed using a BrukerBioSpin Avance III HD spectrometer with a TCI triple-resonance cryogenic probe-head with the basic $^1$H resonance frequency of 500.13 MHz. Three-dimensional (3D) TROSY and WATERGATE versions of HNCACB, HN(CO)CACB, HNCA, HN(CO)CA, HNCO, and HN(CA)CO spectra^66^ were acquired at 310 K for backbone signal assignment of 0.36 mM (as a monomer concentration) [$^2$H, $^{13}$C, $^{15}$N]-SeviL dissolved in 50 mM potassium phosphate buffer (pH 6.8) containing 20 mM NaCl and 7% D_2_O. The spectral widths (the number of total data points) of each spectrum were 24 ppm (2,048) for the $^1$H dimension, and 33 ppm (70) for the $^{15}$N dimension. All spectra were acquired by a non-uniform sampling (NUS) method with 70–90% sampling. Each experiment took about two days. Chemical shift perturbation experiments were performed by recording two-dimensional (2D) $^1$H-$^{15}$N heteronuclear single-quantum correlation (HSQC) spectra of 0.1 mM [$^{15}$N]-SeviL dissolved in the same buffer. NMR spectra were processed using NMRPipe^67^ and SMILE^68^, and signal assignments were performed using the programs MagRO^69^ and NMRView^70^.

STD NMR analysis of the interaction between SeviL and the GM1b saccharide was performed by dialysing protein against water, freeze-drying and redissolving in phosphate-buffered saline (PBS). STD NMR experiments were carried out at 298 K using a Bruker 600 MHz DRX spectrometer fitted with a cryoprobe. The pseudo 2D pulse program stddiff.3 was used. The experiments were recorded as 32000 data points and zero-filled up to 64000 data points prior to processing; the protein resonances were selectively irradiated at 6.5 ppm by a train of Gauss pulses with a length of 50 ms. A 20 ms spin-lock pulse was applied to reduce protein signals. A saturation time of 2 s and a protein:ligand molar ratio of 1:10 were used. The STD effect of a given proton was calculated by using (I0 - Isat)/I0, where (I0 - Isat) is the intensity of the signal in the STD NMR spectrum and I0 is the peak intensity of an unsaturated reference spectrum (off-resonance). The STD signal with the highest intensity, the proton at position 4 of GalNAc (C) residue, was set to 100% and the others were normalised to this peak, allowing ligand epitope mapping. Data acquisition and processing were performed with TOPSPIN 3.2 software. The theoretical STD effects of Sevil-GM1b complex were calculated with the program CORCEMA-ST^71^. The dissociation constant was set in the low millimolar range in accordance with ITC results. The NOE R factor, a normalised root-mean square deviation (RMSD) value, represents a measure of the fit between experimental and theoretical data.

### Hemagglutination assay

Hemagglutination assay was performed in 96-well U-shape plates as described previously^72^. 20 $\upmu$L each protein (10 $\upmu$g/ml) in TBS was mixed with 20 $\upmu$L of a 1% suspension (with TBS; v/v) of trypsinised and glutaraldehyde-fixed rabbit erythrocytes in addition to 20 $\upmu$L of TBS containing 0.25% (w/v) Triton X-100 and 10 mM CaCl_2_. The plate was incubated at room temperature for 1 h, and the formation of a sheet (agglutination-positive) or dot (agglutination-negative) was observed and scored against the lectin titre.

## Supplementary information

Supplementary information 1.

## Data Availability

The final model and structure factors are available from the Protein DataBank with codes 6LF1 and 6LF2.
