# Supplementary material for: The structure of SeviL, a GM1b/asialo-GM1 binding R-type lectin from the mussel Mytilisepta virgata
Source: Sci Rep. 2020 Dec 16;10:22102. doi: 10.1038/s41598-020-78926-7 (PMC7744527; doi:10.1038/s41598-020-78926-7)
Supplement: Supplementary file 1 — Supplementary information 1. [file 41598_2020_78926_MOESM1_ESM.pdf]

The structure of SeviL, a GM1b/asialo-GM1 binding R-type lectin from the mussel *Mytilisepta virgata*

Kenichi Kamata[1], Kenji Mizutani[1], Katsuya Takahashi[1], Roberta Marchetti[2], Alba Silipo[2], Christine Addy[1], Sam-Yong Park[1], Yuki Fujii[3], Hideaki Fujita[3], Tsuyoshi Konuma[1], Takahisa Ikegami[1], Yasuhiro Ozeki[4], and Jeremy R. H. Tame[1,*]

[1]Graduate School of Medical Life Science, Yokohama City University, 1-7-29 Suehiro, Yokohama, Kanagawa 230-0045, Japan
[2]Department of Chemical Sciences, Università di Napoli Federico II, Via Cintia 4, I-80126, Naples, Italy
[3]Department of Pharmacy, Graduate School of Pharmaceutical Science, Nagasaki International University, 2825-7 Huis Ten Bosch, Sasebo, Nagasaki 859-3298, Japan
[4]Laboratory of Glycobiology and Marine Biochemistry, Graduate School of NanoBio Sciences, Yokohama City University, 22-2, Seto, Yokohama, Kanagawa 236-0027, Japan
[*]jtame@yokohama-cu.ac.jp

Supplementary Information

Experimental

Cloning

A synthetic gene encoding SeviL was designed with flanking NdeI and BamH1 restriction sites. Codon optimization was performed using an in-house back-translation tool, and the gene was synthesised by IDT. The designed DNA sequence was excised from the supplied plasmid DNA and inserted into appropriately cut pET28, using T4 DNA ligase (Wako) at room temperature for 1 h. The ligation mixture was used to transform *E. coli* DH5α, and pET28-SeviL was prepared from cultures using QIAprep (Qiagen). This vector directs expression of full-length SeviL carrying a thrombin-cleavable hexa-histidine tag at the N-terminus. The final purified protein product, after tag removal, has a sequence beginning GSHMA.

Expression and purification

E. coli BL21(DE3) cells were transformed with the plasmid pET28-SeviL. Colonies were transferred to LB medium containing kanamycin (20 μg/mL), and grown with shaking at 310 K. When the culture O.D.600 reached ~0.6, SeviL was expressed by adding IPTG (final concentration 0.5 mM), and growth was continued for 3 h at the same temperature. The cells were collected by centrifugation at 3000×g at 277 K for 30 min. The pellet was suspended in 0.1 M Tris-HCl, pH 8.0, 0.15 M NaCl and sonicated on ice. The lysate was centrifuged at 38000×g at 277 K for 45 min.

The supernatant was loaded onto 5 mL nickel-sepharose column (GE), and washed with 0.1 M Tris-HCl, pH 8.0, 0.15 M NaCl, and after washing, eluted with 0.1 M Tris-HCl, pH 8.0, 0.15 M NaCl, 0.25 M imidazole. The fractions including protein were collected and digested with thrombin at 277 K overnight during dialysis with wash buffer. The protein was reloaded onto the nickel-sepharose column and the flow-through was collected. The protein was dialyzed into 20 mM Tris-HCl, pH 7.4, 20 mM NaCl before loading onto a Q-sepharose column (GE) and elution with a gradient to 20 mM Tris-HCl, pH 7.4, 1 M NaCl. The protein fraction was concentrated to 2 mL and loaded onto a size-exclusion column with 20 mM Tris-HCl, pH 7.4, 0.1 M NaCl. SeviL mutants were expressed and purified by the same method. The pellet was suspended in 100 mM Tris HCl pH 8.0, 0.15 M NaCl, 20 mM imidazole and then lysed by sonication on ice. The lysate was centrifuged at 38,000×g at 277 K for 50 min. The supernatant solution was loaded onto a 10 ml volume nickel-sepharose column (GE Healthcare) equilibrated with 100 mM Tris HCl pH 8.0, 0.15 M NaCl, 20 mM imidazole, and after washing, eluted with 20 mM Tris HCl pH 8.0, 500 mM imidazole, 150 mM NaCl. The SeviL-containing fractions were collected and digested with thrombin overnight at 277 K during dialysis into 20 mM Tris HCl pH 7.4, 50 mM NaCl. The protease:SeviL ratio was 1:200. The protein was re-loaded onto the washed nickel-sepharose column and eluted with 20 mM Tris HCl pH 7.4, 20 mM NaCl. The pooled fractions containing SeviL were dialyzed into 20 mM Tris HCl pH 7.4, 50 mM NaCl before loading onto a Q-sepharose column (GE) equilibrated with the same buffer, and eluted with a gradient to 1 M NaCl. The pooled protein fractions were concentrated to 10 mg/ml using Amicon centrifugal filter units (Millipore).

Contacts between subunits of the SeviL dimer				
A chain		B chain		Distance (Å)
12(GLN)	OE1	12(GLN)	NE2	2.9
12(GLN)	NE2	36(MET)	SD	3.5
		12(GLN)	OE1	2.9
14(ARG)	O	36(MET)	N	2.9
15(GLU)	O	35(LYS)	CB	3.3
		35(LYS)	CA	3.4
34(GLY)	O	16(ASN)	C	3.4
		17(GLY)	N	3.3
35(LYS)	CA	14(ARG)	O	3.5
		15(GLU)	O	3.4
35(LYS)	CB	15(GLU)	O	3.3
35(LYS)	CG	14(ARG)	O	3.5
35(LYS)	CE	15(GLU)	OE1	3.5
35(LYS)	NZ	15(GLU)	OE1	3.0
36(MET)	N	14(ARG)	O	2.8
36(MET)	SD	12(GLN)	NE2	3.4
91(GLY)	CA	128(ALA)	O	3.4
126(PHE)	CD1	126(PHE)	CG	3.5

Table 1. Distances no greater than 3.5 Å between the two subunits of the complex.

Hydrogen bonds formed between SeviL and asialo-GM1				
SeviL		asialo-GM1		Distance (Å)
28(ASP)	OD2	2(GAL)	O6	2.7
40(GLN)	NE2	3(NGA)	O6	2.8
39(ASP)	OD1	3(NGA)	O6	2.7
26(ARG)	NH1	3(NGA)	O4	3.0
26(ARG)	NH2	3(NGA)	O4	2.9
39(ASP)	OD2	3(NGA)	O4	2.5
26(ARG)	NH1	3(NGA)	O3	3.4
39(ASP)	N	4(GAL)	O6	3.1

Table 2. Distances no greater than 3.5 Å between the atoms of the protein and saccharide, in a suitable orientation for hydrogen bond formation.

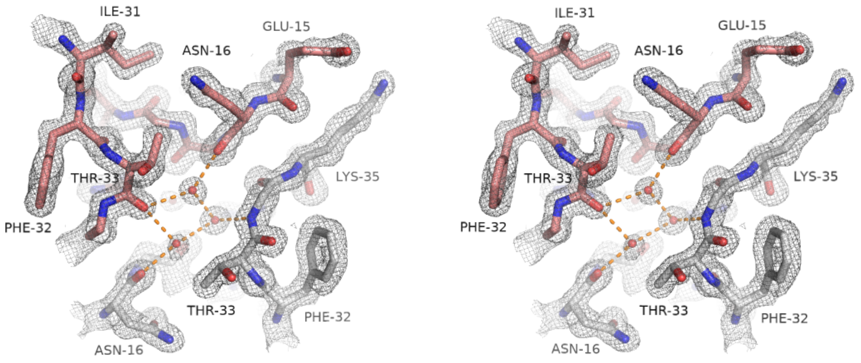

Figure 1. A stereo view of the $2mF_o - DF_c$ electron density map of the liganded SeviL structure, contoured at 1 σ, showing the dimer contact near Thr 33, where several water molecules are deeply buried between the protein subunits. The carbonyl of the two copies of Thr 33 have different orientations relative to the rest of the subunit. The two protein molecules have carbon atoms coloured either white or pink.

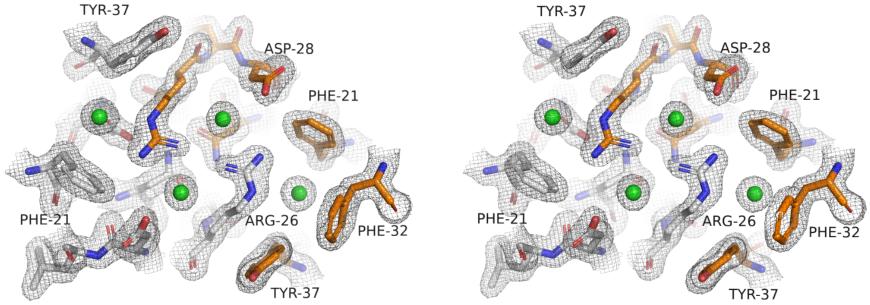

Figure 2. A stereo view of the $2mF_o - DF_c$ electron density map of the apo-SeviL structure, contoured at 1 σ, showing the crystal contact formed by Arg 26, which sits in the ligand binding site of a neighboring protein molecule. The two protein molecules have carbon atoms coloured either white or orange. Chloride ions are shown as green spheres.

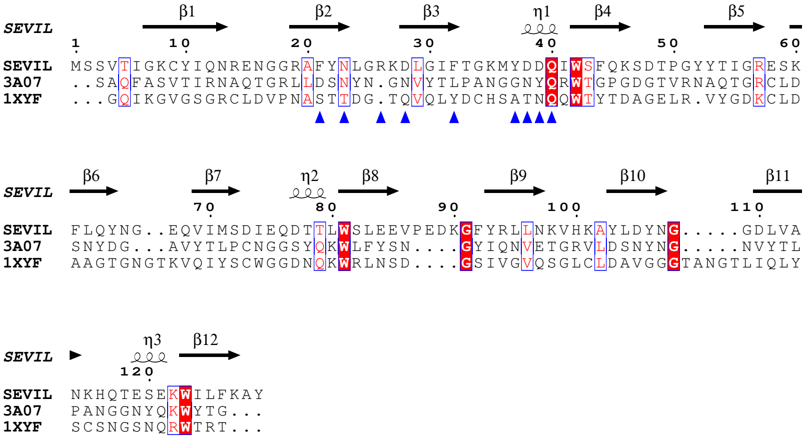

Figure 3. A sequence alignment of SeviL with actinohivin (PDB model 3A07) and the xylan binding domain of an endo-xylanase (PDB model 1XYF). The residues of SeviL that contact the saccharide ligand are indicated with blue triangles, showing the three proteins are unconserved in this region. β-strands of SeviL are shown as arrows, and helical structure is indicated with a looping line, over the sequence. Conserved residues are shown in white on red, and similar side-chains are shown in red. This figure was produced with Espript (http://espript.ibcp.fr).

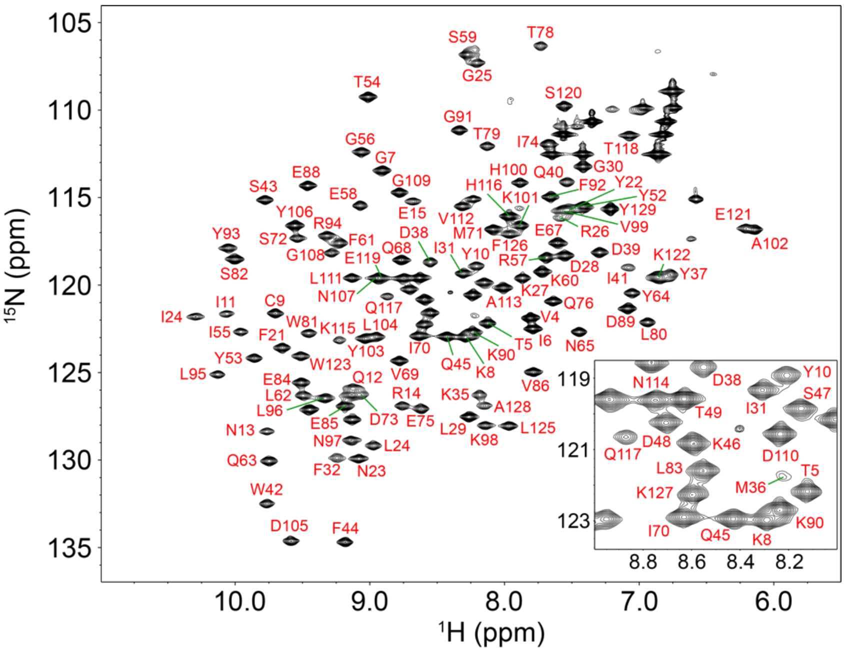

Figure 4. The complete assignment of the ^{1}H-^{15}N HSQC NMR spectrum of SeviL.

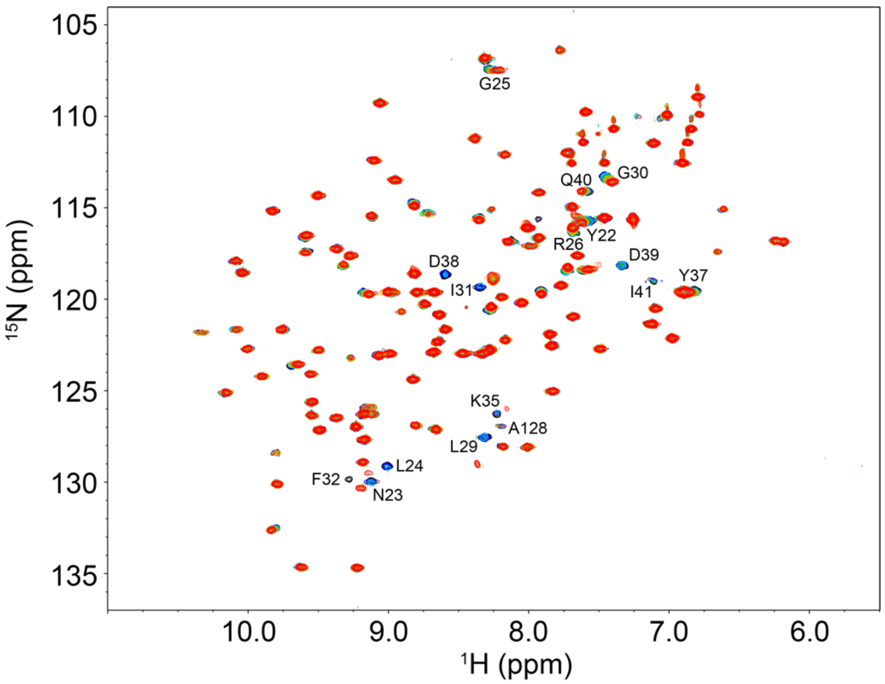

Figure 5. Overlay of 2D ^{1}H-^{15}N HSQC spectra of SeviL, showing the residues affected by the presence of asialo-GM1. Spectra are coloured by concentration of asialo-GM1 saccharide: 0 mM (black), 0.05 mM (blue), 0.1 mM (cyan), 0.2 mM (green), 0.3 mM (yellow), 0.5 mM (orange) and 1 mM (red).

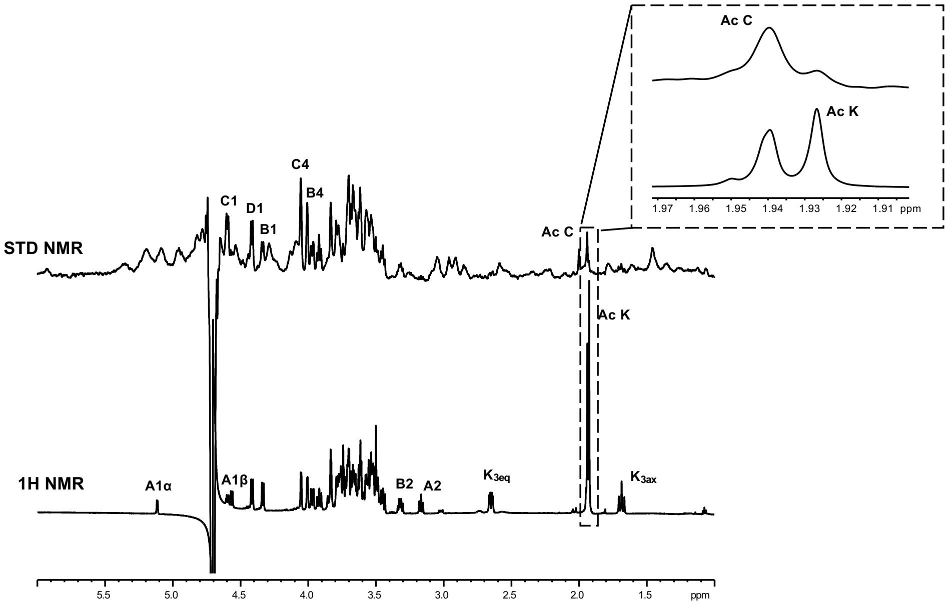

Figure 6. STD NMR spectrum of the 1:10 mixture Sevil:GM1b. Analysis of isolated signals in the STD NMR spectrum clearly revealed that the glucose residue (BGC1, A) was not directly involved in the interaction with the protein. No significant STD NMR enhancements were observed for the anomeric proton H1 of the glucose residue nor for its proton at position 2. Similarly the sialic acid residue (SIA, K) did not contribute to binding as clearly indicated, for example, by the absence of STD signal from its acetamide moiety at 1.93 ppm. A slight but detectable STD signal was observed belonging to the acetyl group of the GalNAc residue (NAG3, C).

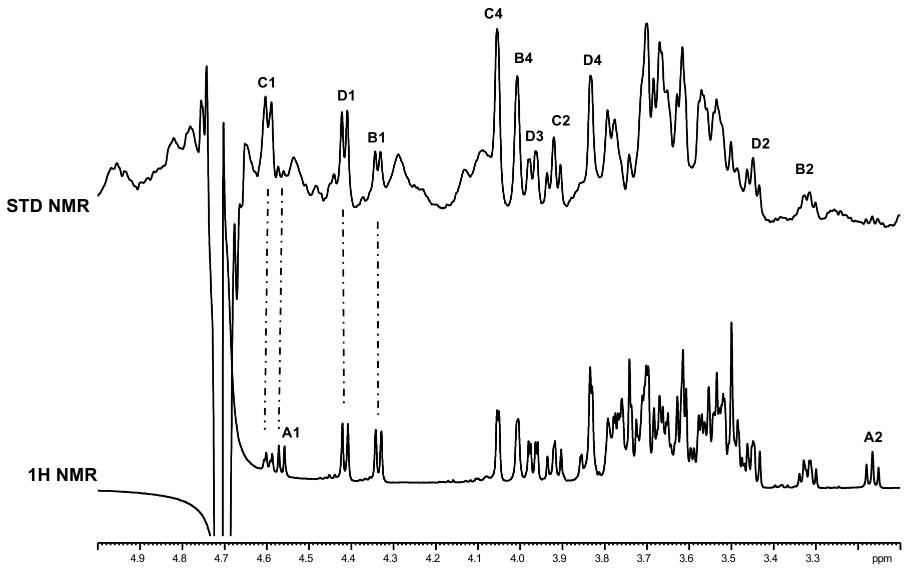

Figure 7. Close-up of the STD NMR spectrum highlighting the assignment of the signals.

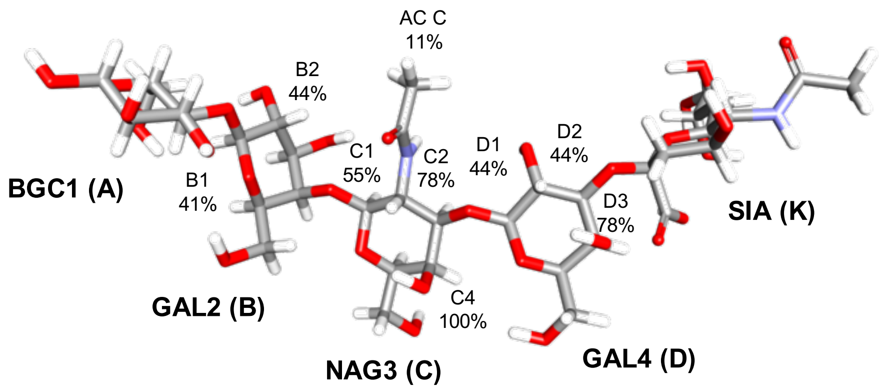

Figure 8. Model of GM1b saccharide showing the derived STD values for protons close to SeviL in the complex. The percentage of STD effects for these protons was derived and normalized to the highest STD signal. Residues of the saccharide are labelled from A to K as shown.

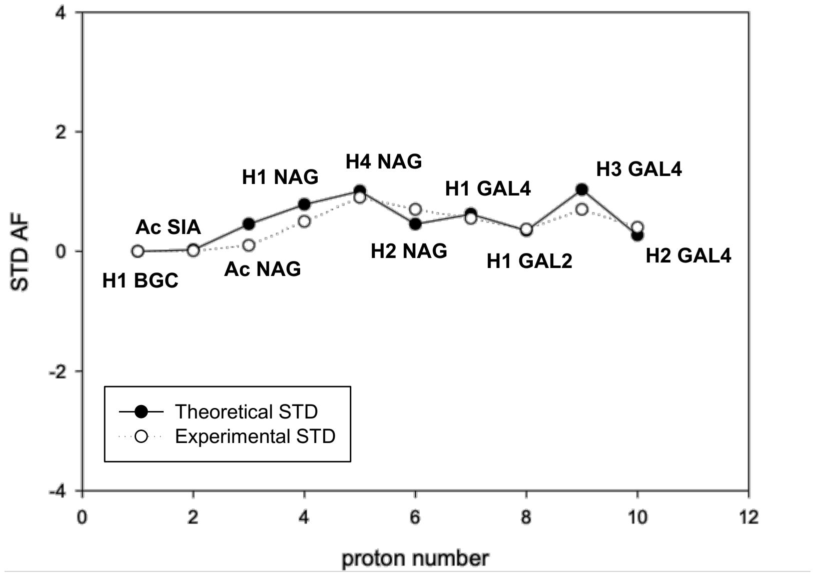

Figure 9. Comparison between experimental (dashed line) and theoretical (solid line) STD data for the model of GM1b in the presence of Sevil. STD AF stands for STD Amplification Factor. The interaction of Sevil with GM1b was characterized by molecular modeling and computational studies based on the X-ray structure, further confirming the crucial protein-ligand interactions and indicating that the sialic acid pointed away from the protein surface. CORCEMA-ST (Complete relaxation and conformational exchange matrix analysis of saturation transfer) was used to predict theoretical STD effects from the model and compare them to those experimentally observed. Fitting the experimental and calculated STD values resulted in an NOE R factor of 0.3.

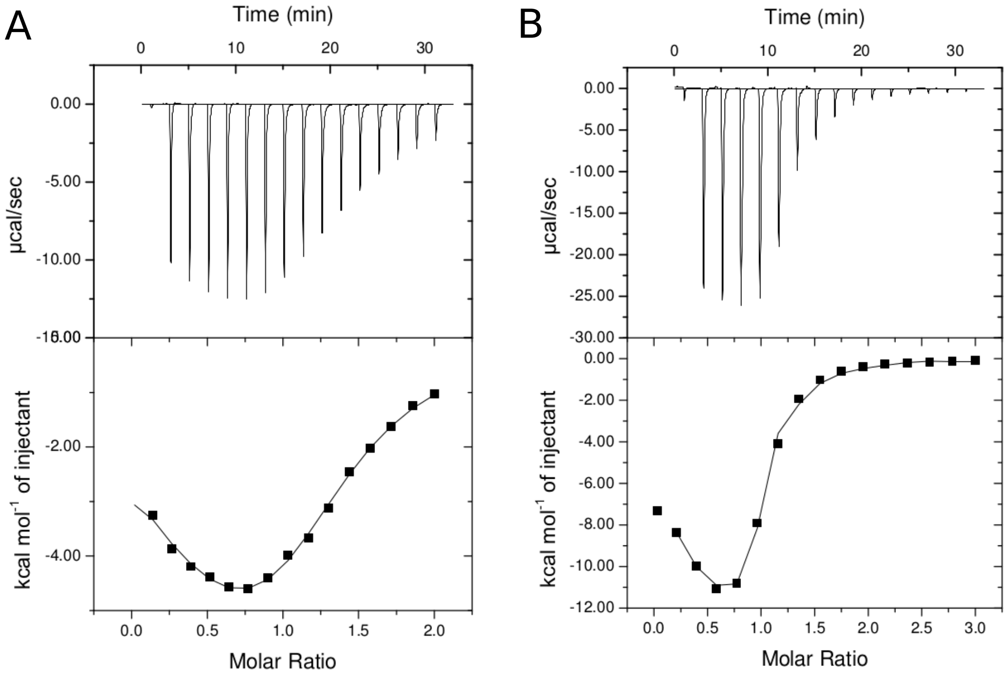

Figure 10. ITC experiments carried out with higher concentrations of protein and ligand. (A) 1 mM SeviL titrated with 10 mM asialoGM1 saccharide. (B) 0.8 mM SeviL titrated with 12 mM GM1b saccharide. In both cases the shape of the thermogram was reproducible, with the enthalpy of binding having greater magnitude for the longer ligand. The marked increase in heat release with progress of the titration indicates site-site interaction or non-ideal effects. No evidence of allosteric interaction is found in the model, suggesting that at high concentration the sugar ligands form non-ideal solutions.

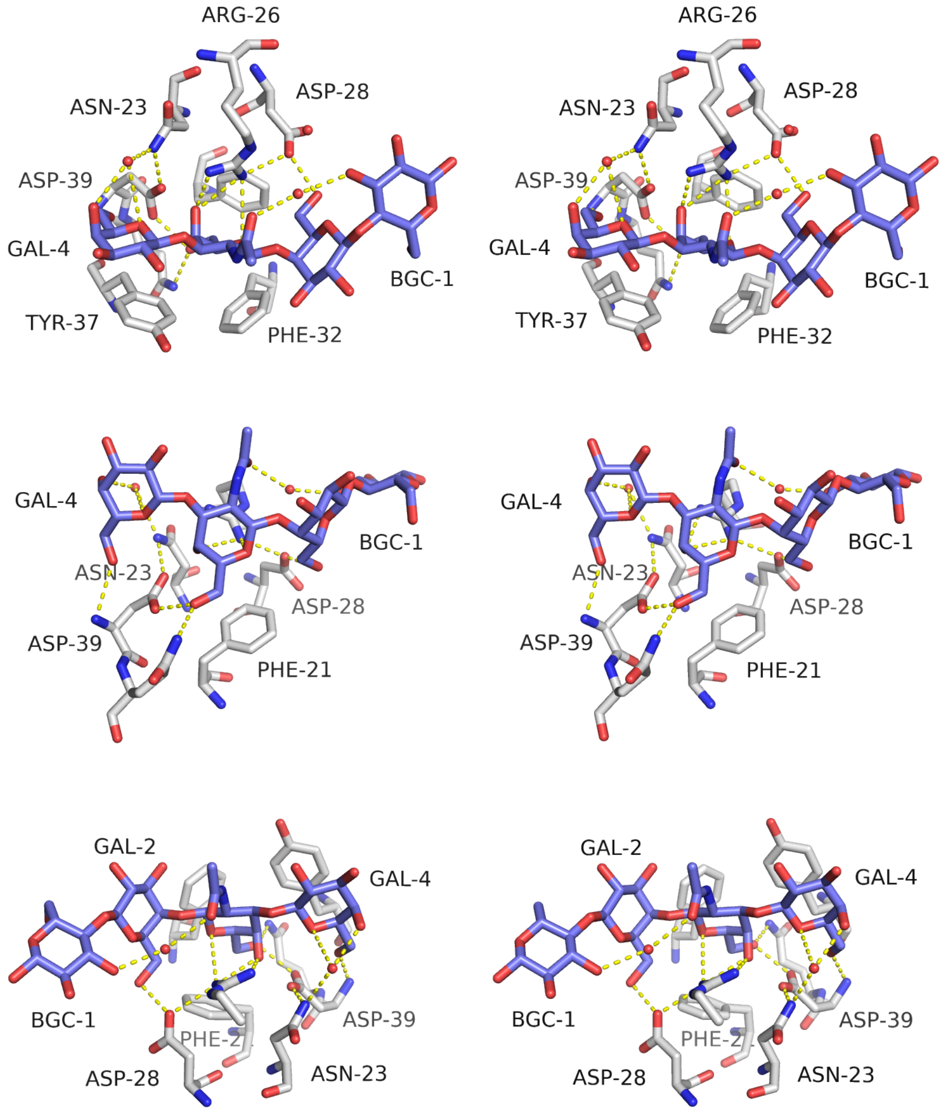

Figure 11. Three different stereo views of the liganded SeviL structure. The protein molecule has carbon atoms coloured white, and asialo-GM1 has carbon atoms coloured purple. Hydrogen bonds between the two are shown as dotted yellow lines.
